# g-FLUA2H: a web-based application to study the dynamics of animal-to-human mutation transmission for influenza viruses

**DOI:** 10.1186/1755-8794-8-S4-S5

**Published:** 2015-12-09

**Authors:** Muhammad Farhan Sjaugi, Swan Tan, Hadia Syahirah Abd Raman, Wan Ching Lim, Nik Elena Nik Mohamed, J Thomas August, Asif M Khan

**Affiliations:** 1 Centre for Bioinformatics, Perdana University, Jalan MAEPS Perdana, 43400 Serdang, Selangor, Malaysia; 2 Department of Pharmacology and Molecular Sciences, The Johns Hopkins University School of Medicine, 725 North Wolfe Street, 21205 Baltimore, MD, USA; 3 Graduate School of Medicine, Perdana University, Jalan MAEPS Perdana, 43400 Serdang, Selangor, Malaysia

**Keywords:** influenza viruses, animal-to-human transmission, mutation

## Abstract

g-FLUA2H is a web-based application focused on the analysis of the dynamics of influenza virus animal-to-human (A2H) mutation transmissions. The application only requires the viral protein sequences from both the animal and human host populations as input datasets. The comparative analyses between the co-aligned sequences of the two viral populations is based on a sliding window approach of size nine for statistical significance and data application to the major histocompatibility complex (MHC) and T-cell receptor (TCR) immune response mechanisms. The sequences at each of the aligned overlapping nonamer positions for the respective virus hosts are classified as four patterns of characteristic diversity motifs, as a basis for quantitative analyses: (i) "index", the most prevalent sequence; (ii) "major" variant, the second most common sequence and the single most prevalent variant of the index, with at least one amino acid mutation; (iii) "minor" variants, multiple different sequences, each with an incidence (percent occurrence) less than that of the major variant; and (iv) "unique" variants, each with only one occurrence in the alignment. The diversity motifs and their incidences at each of the nonamer positions allow evaluation of the mutation transmission dynamics and selectivity of the viral sequences in relation to the animal and the human hosts. g-FLUA2H is facilitated by a grid back-end for parallel processing of large sequence datasets. The web-application is publicly available at http://bioinfo.perdanauniversity.edu.my/g-FLUA2H. It can be used for a detailed characterization of the composition and incidence of mutations present in the proteomes of influenza viruses from animal and human host populations, for a better understanding of host tropism.

## Introduction

Influenza has a history as one of the world's most serious pathogens, with yearly regional infections and episodic global pandemics. Influenza viruses belong to the *Orthomyxoviridae *family of RNA viruses and occur as three major types: influenza A, infectious in birds and some mammals, including humans [1]; influenza B, a genus that infects only humans and seals [2]; and influenza C, a rare type that is known to have infected human and pigs [3]. Proliferation of influenza A is predominantly in avian hosts with very rapid mutation, resulting in a "quasispecies" [4], a vast number of viruses that are genetically related but differ in the amino acid sequences of the viral proteins. These mutations are a classic example of Darwinian evolution [5]; they occur in a random fashion and the variant viruses that have the best genetically endowed combination of efficient infection, rapid replication, and greatest survival become the dominant populations. The survival properties include virus escape from the immune responses of humans previously infected or immunized with an earlier virus strain.

Human infections by influenza A viruses commonly occur yearly, with a seasonal peak incidence [6], usually as a mild disease, but for some, as a more severe illness that may be fatal. A major complication is the occurrence of global pandemics resulting from the emergence of highly infectious subtypes of the virus, particularly those capable of human-to-human transmission. The history of influenza pandemics began with the H1N1 "Spanish Flu" strain of 1918-1919 that killed an estimated fifty million people [7]. This was followed by other less severe strains, the H2N2 "Asian influenza" of 1957-1958, H3N2 "Hong Kong flu" of 1968-1969, and H5N1 "bird flu" in 2006-2007, and recently the H1N1 "swine flu" of 2009-2010. It thus appears inevitable that, without means to prevent influenza infection, another pandemic will occur within the foreseeable future. It is simply a matter of chance that the mutations responsible for the infectivity and pathogenicity of a particular influenza virus in animals does not include the ability to efficiently infect humans with human-to-human transmission. If a strain comparable to that of the 1918 pandemic was to occur, the global consequences are inconceivable.

Sequence change (mutation) events can transform an animal-origin virus into a human virus, with varying levels of fitness to survive in the new host [7]. A number of mutations that facilitate the transmission from animal to human hosts have been described for the influenza A viral proteome. For example, specific amino acid substitutions can alter the host receptor binding site and specificity of hemagglutinin (HA) protein, a major determinant of host tropism, for preference from avian to human sialic acid linkages [1]. Additionally, a single amino acid change at position 627 of the viral polymerase complex subunit PB2 was found to enhance replication in human host [8]. There remains, however, the need for a comprehensive identification of sequence changes that allow or enhance human infection, with potential applications to influenza virus surveillance and possibly to prevention or treatment of human infection.

A number of computational models have been reported that discriminate between animal and human influenza A viruses based on molecular patterns in protein sequences [9-12] and genomic signatures [13]. Although these models are potentially useful for predicting interspecies transmission of influenza viruses, there is still a great difficulty in deciphering adaptation of viruses that show mixed signatures of both animal and human hosts. There remains the need for a greater understanding of the viral sequence diversity and the dynamics of sequence change, including the composition and incidence of mutations between the viral host populations.

Herein, we present g-FLUA2H, a web-based application, with a grid-backend, to analyze the dynamics of animal-to-human (A2H) mutation transmission for influenza viruses. The application only requires the viral protein sequences from both the animal and human host populations as input datasets. g-FLUA2H is facilitated by a grid back-end to manage large datasets, as there are currently more than 500,000 sequences of influenza A virus reported in the public Influenza Research Database (IRD; http://www.fludb.org/). g-FLUA2H is publicly available at http://bioinfo.perdanauniversity.edu.my/g-FLUA2H.

## System description

**Figure 1:**
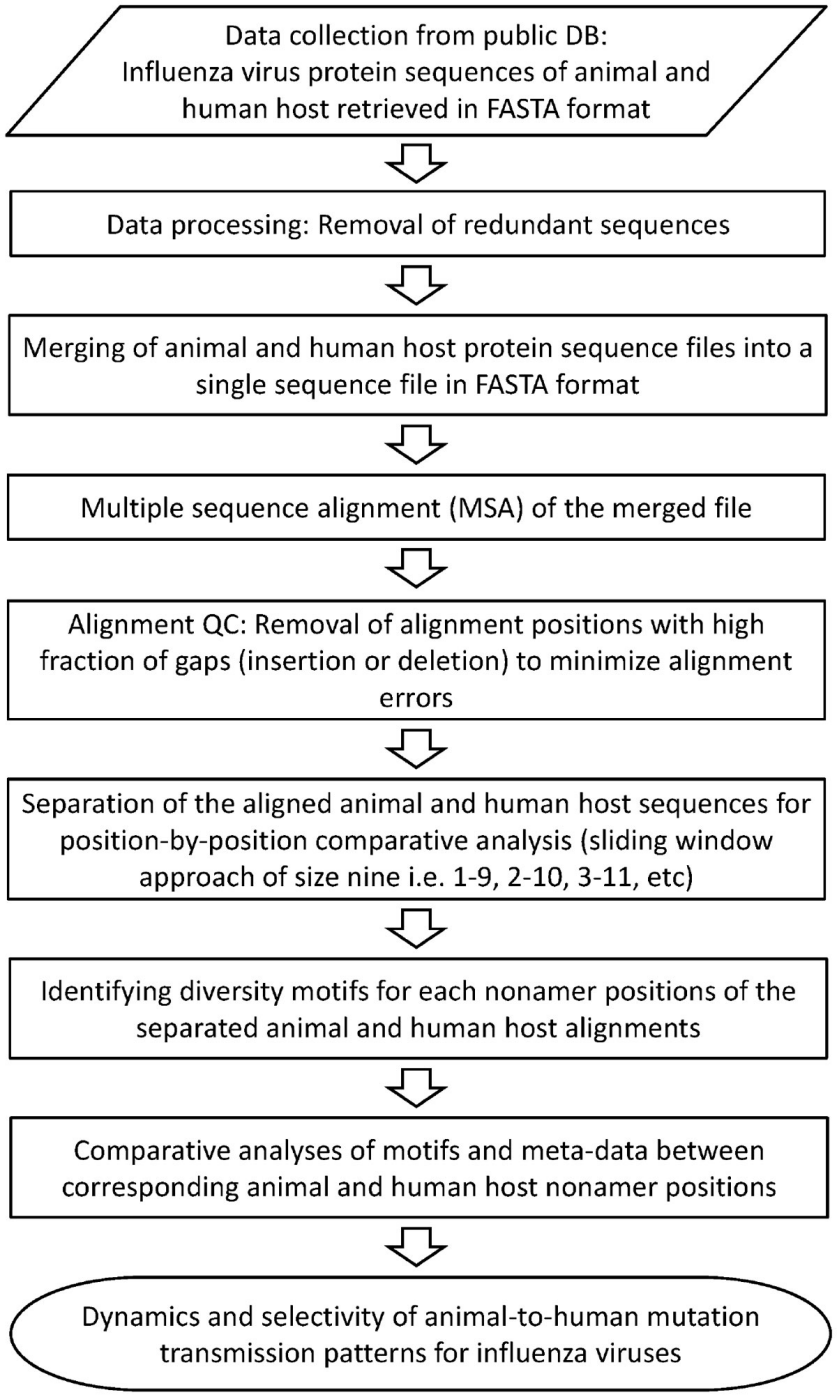
**Schematic workflow of the methodology employed by g-FLUA2H**.

Figure 1 provides a schematic workflow summarizing the methodology employed by g-FLUA2H. Input protein sequences, in FASTA format, can be downloaded from the publicly available Influenza Research Database (IRD; http://www.fludb.org) for all worldwide, recorded animal and human influenza viruses. There is a specific format for the FASTA file header that needs to be followed: "> Strain name information | Accession number | Protein name" where the strain name is essential and follows the influenza nomenclature by WHO in the format "Antigenic type/Host/Location of sampling/Isolate ID code/Year of sampling". There is no specific format for accession number and protein name, both of which are optional. An example of a FASTA header format is "> A/blue_winged_teal/Ohio/566/2006 | A7IRT3 | HA". Instructions on how to download sequences from the FluDB in this header format are provided on the help page of g-FLUA2H.

Both full-length and incomplete partial sequences are considered for the analyses. Partial sequences are included because they provide additional data, however, they can be a source of spurious multiple sequence alignment, which require manual editing to correct the misalignment. Duplicate sequences for each protein are removed if present. The non-redundant sequences of each protein from both the animal and human hosts of the virus are then merged for a co-alignment by use of a local copy of MAFFT (obtained from http://mafft.cbrc.jp/alignment/software/); MAFFT is one of the most accurate multiple sequence alignment methods currently available [14]. The co-alignment provides for a unified alignment position, allowing comparison between the two host populations. Users are provided with parameters to disable the removal of redundant sequences and to remove alignment positions with a high fraction of gaps (insertions or deletions), considered to be of low statistical support. Additionally, users are provided with the option to upload manually edited co-alignments as input, in TALN format, if necessary. The sequences of each host population are eventually separated from the co-alignment for a position-by-position comparative analyses.

The comparative analyses is based on a sliding window approach of size nine (1-9, 2-10, 3-11, *etc*) on the separated alignments [15-17]. Each of the aligned overlapping nonamer (9-mer) positions represent a possible antigenic core binding domain for human leukocyte antigen (HLA; human MHC) molecules and T-cell receptors [16,17]; this assumption is based on the fact that there are large array of HLAs with different binding specificities in the human population [17]. Further, the repeated associations of each amino acid in a moving, overlapping 9-mer window can facilitate the detection of possible sequencing errors. The sequences at each of the 9-mer positions are classified as four patterns of characteristic diversity motifs that we previously defined in Hu, Y. *et al*. [18] and discussed the biological implications. In brief, the motifs are: (i) "index", the most prevalent sequence; (ii) "major" variant, the second most common sequence and the single most prevalent variant of the index, with at least one amino acid mutation; (iii) "minor" variants, multiple different sequences, each with an incidence (percent occurrence) less than that of the major variant; and (iv) "unique" variants, each observed only once. The quantification of the diversity motifs is detailed in [18] and also described on the g-FLUA2H help page (see Section D). The diversity motifs and their incidences at each of the nonamer positions allow evaluation of the mutation transmission dynamics and selectivity of the sequences in relation to the animal or human hosts. Distinct sequences of any motif that contain gaps (-) or any of the unresolved characters, including B (asparagine or aspartic acid), J (leucine or Isoleucine), X (unspecified or unknown amino acid), and Z (glutamine or glutamic acid) are excluded from all analyses.

**Figure 2:**
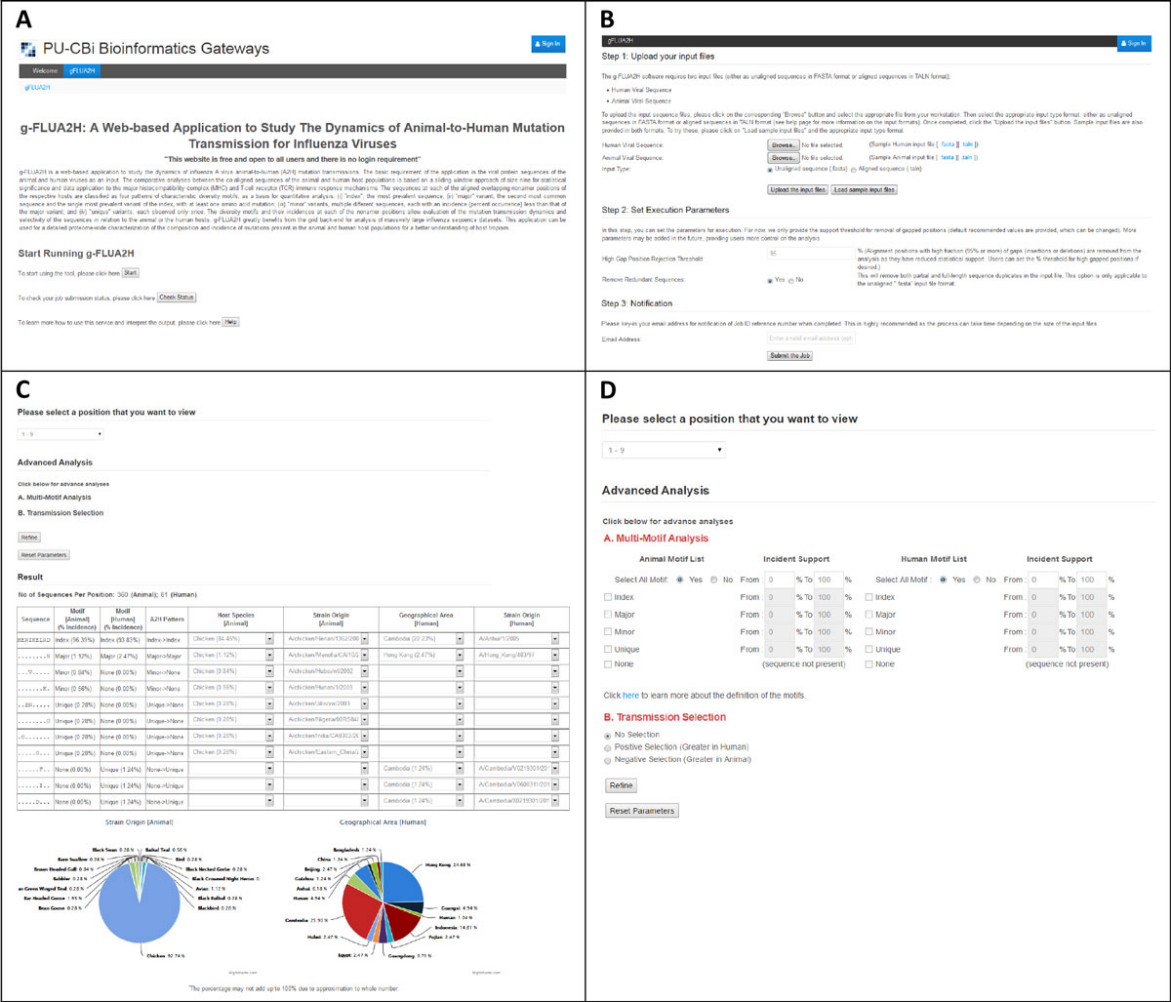
**Snapshots of selected g-FLUA2H features**. **A.** Homepage interface of g-FLUA2H, which includes a brief description of the application and links to start an analysis, check job status and get more information from the help page. **B**. Web-interface to upload input sequence files, parameters to disable the removal of redundant sequences and to remove alignment positions with a high fraction of gaps (insertions or deletions), and an option to receive email notification when the job submission is complete. **C**. A sample output page, providing users with a dropdown menu listing all the nonamer positions to view motif data compared between the animal and human hosts of the virus. The selection of a given nonamer position presents users with a detailed information on the distinct nonamer sequences at the position for both hosts, such as their motif classifications and incidences (% occurrence), amino acid substitutions relative to the index, strain name of origin virus in animal and human hosts, host species of the animal host, and the geographical distribution of the human host. **D**. Options for advanced analyses on the results, such as multi-motif analysis and transmission selection where users can analyse for any combination of the diversity motifs and incidences between the hosts, and filter for positive (the incidence of the corresponding motif is greater in human host) or negative selections (incidence of motif is greater in animal host).

**Figure 3:**
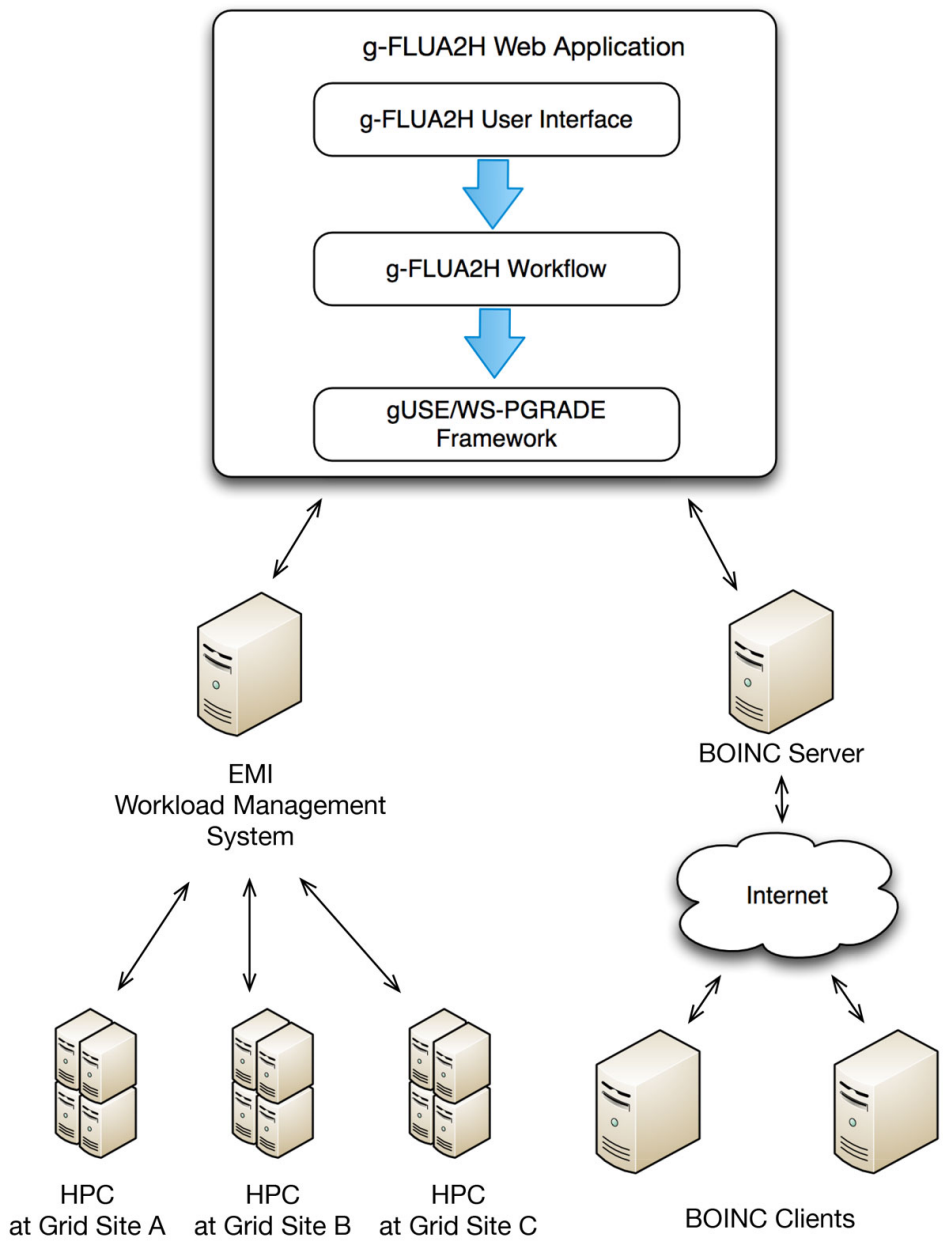
**The grid-backend of g-FLUA2H**. The user interface of the web application allows input of data which then undergoes a series of steps as part of the data processing and analysis workflow, built on top of the gUSE/WS-PGRADE framework. Depending on the computing load, the framework manages distribution of jobs. Compute intensive executions are handled by sending jobs to a computing cluster, such as HPC/Grid; in this case to the Academic Grid Malaysia Infrastructure (http://academicgrid.my), which is based on the EMI middleware (http://www.eu-emi.eu). Compute less-intensive executions are sent to the distributed Desktop Grid (http://desktopgridfederation.org), based on BOINC setup (https://boinc.berkeley.edu). Abbreviations: HPC, high performance computing; gUSE/WS-PGRADE, Grid and cloud User Support Environment/Web Services Parallel Grid Runtime and Developer Environment; EMI, European Middleware Initiative; BOINC, Berkeley Open Infrastructure for Network Computing.

The output page of g-FLUA2H provides users with a dropdown menu listing all the nonamer positions to view motif data compared between the animal and human hosts of the virus. The selection of a given nonamer position presents users with a detailed information on the distinct nonamer sequences at the position for both hosts, such as their motif classifications and incidences, amino acid substitutions relative to the index, strain name of origin virus in animal and human hosts, host species of the animal host, and the geographical distribution of the human host. Advanced functionalities include multi-motif analysis and transmission selection where users can analyse for any combination of the diversity motifs and incidences between the hosts, and filter for positive (the incidence of the corresponding motif is greater in human host) or negative selections (incidence of motif is greater in animal host). Snapshots of the key features of g-FLUA2H are provided in Figure 2.

g-FLUA2H was built on top of the gUSE/WS-PGRADE framework [19-23], a grid-backend with multiple CPU cores (several hundreds). This was implemented not as a key distinguishing feature, but for practical purpose to allow ease of handling large datasets, and thus reducing computation time. The g-FLUA2H pipeline employs a combination of parallel compute intensive and distributed compute less-intensive executions (Figure 3).

## g-FLUA2H Application: animal-to-human mutation patterns of amino acid substitution E627K in influenza A H5N1 and H7N9 subtypes

**Figure 4:**
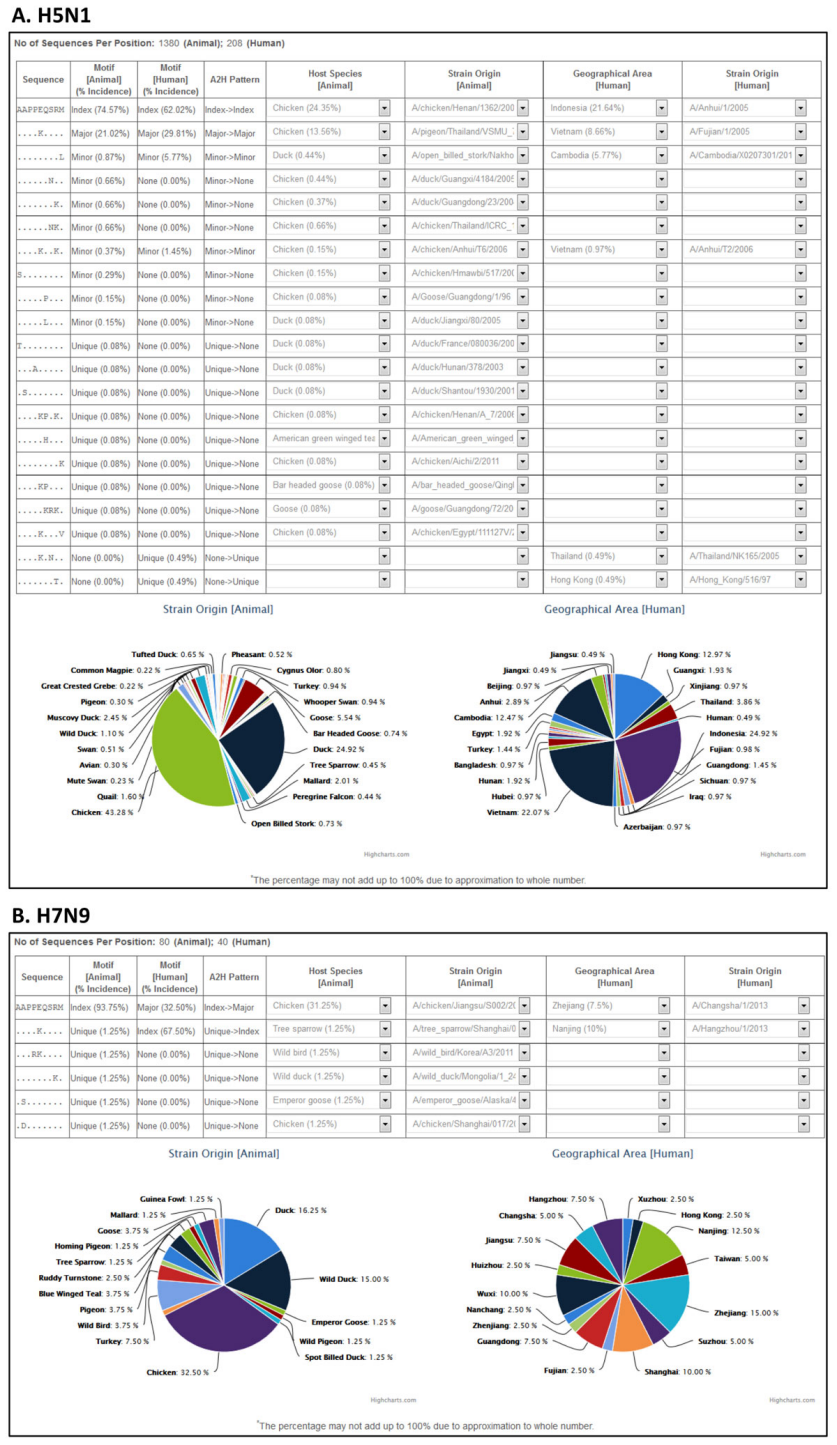
**g-FLUA2H output for a representative PB2 nonamer position containing the E627K substitution, reported to be crucial for host tropism**. The representative position for H5N1 (panel A) and H7N9 (panel B) are the same, 623-631. The output provides detailed information on the distinct nonamer sequences reported at the position for both animal and human hosts of the virus, such as their motif classifications and incidences (% occurrence), amino acid substitutions relative to the index, strain name of origin virus in animal and human hosts, host species of the animal host, and the geographical distribution of the human host. The dropdown menu for each of the distinct nonamer sequences shows the animal species origin, strain names of origin virus in animal and human hosts, and geographical locations of the viral isolate for the human host. The pie-charts show the frequency distribution of the animal host species origin and the geographical location of the human isolates for the nonamer position as a whole, covering all the distinct sequences.

The amino acid substitution E627K in PB2 of the viral polymerase complex has been reported to be crucial for host tropism. Glutamic acid (E) is found at position 627 in most of the avian strains, whereas replacing the amino acid with lysine (K) enhances viral replication in humans [8,24-28]. The dynamics of this substitution between the animal (avian) and human populations was analyzed and compared by use of g-FLUA2H for H5N1 and H7N9 subtypes, over the time period and the geographical range of the reported data in IRD (Figure 4). There were 2402 (avian: 2130, human: 272) and 127 (avian: 84, human: 43) PB2 sequences of H5N1 and H7N9 reported in IRD as of May 2015 and April 2014, respectively.

g-FLUA2H sample output for a representative nonamer position of H5N1 PB2 that contained this mutation is shown in Figure 4A. At this position (623-631; alignment numbering), there were 21 distinct nonamer sequences representing the reported populations of both avian and human viruses. Both populations contained the same index sequence AAPP**E**QSRM, present in ~75% of the 1380 avian and ~62% of the 208 human virus sequences reported in the public database for this position. Chicken (~24%) was the dominant avian source species for this shared index sequence, followed by duck (~20%, dropdown menu; number not shown in Figure 4A) and many other host species (~< 4% each). The human strains containing this sequence were isolated primarily in Indonesia (~22%) and from many other geographical regions in Asia.

The other dominant sequence AAPP**K**QSRM contained a mutation relative to the index that corresponded to the E627K substitution. This sequence was classified as the major variant of the avian index, present in ~21% of the avian sequences and corresponded to the human major variant, present in ~30% of the human sequences. Chicken (~14%) was also the dominant avian source for this sequence. The human strains containing this sequence were isolated primarily from Vietnam (~9%). Although the E627K substitution has been reported to be important for host tropism, it is clear here that a large majority of H5N1 viruses that infected human did not possess this mutation. Two of the six distinct nonamer sequences of human viruses at this position were not present in the avian viruses, suggesting human specific mutations. Similarly, as many as 15 distinct nonamer sequences appeared to be avian specific, non-existent in the reported human viruses.

In comparison, the corresponding aligned H7N9 PB2 position (623-631) contained only six distinct nonamer sequences representing both the avian and human viruses (Figure 4B), and all but two (A624D; P626R & E627K) matched to H5N1. However, unlike H5N1, the avian and human viruses did not share the index sequence. The avian index sequence AAPP**E**QSRM, present in ~94% of the 80 sequences reported in the public database for this position, was the same as the human major variant, present in ~33% of the 40 human sequences reported for this position. Chicken (~31%) was the dominant avian source species for this sequence, followed by duck (~16%), wild duck (~14%), and many other host species (< 8% each) (dropdown menu; numbers not shown in Figure 4B). The human virus strains containing this sequence were primarily isolated from various cities in mainland China. Notably, the index sequence of the human viruses (~68%), which contained the E627K substitution (AAPP**K**QSRM), was found in the avian host population solely as a unique sequence (~1%) in the Tree sparrow species. The other four distinct nonamers were unique sequences (~1% each) and appeared to be avian specific, not present in the human viruses. There was no report of human specific mutations for H7N9 at this position, possibly due to the short history of the virus in the human host.

In summary, the nonamer sequence (AAPP**E**QSRM) without the E627K substitution was conserved across both avian H5N1 and H7N9 viruses as the index sequences, with chicken as the dominant avian source species. However, the motif transmission pattern to human differed; it remained as index in human H5N1 viruses, but was a major variant in human H7N9 viruses. The sequence containing the E627K substitution (AAPP**K**QSRM) was a major variant in both the avian and human H5N1 viruses, and did not appear to be required by all human viruses. In contrast, this substitution was only seen once (a unique variant) in the reported avian H7N9 viruses, but was predominantly observed in the human viruses as the index, possibly indicating a role in host tropism. Furthermore, the analysis identified human specific mutations for H5N1, suggesting further adaptation in the human host. These results merit further investigation (Tan, S. *et al*., manuscript in preparation), and the small number of sequences for the reported human viruses, particularly for H7N9, should be treated as a caveat, with the possibility of sampling bias.

## Conclusion

There is a rich metadata of information that can be obtained from a comparative analysis of animal and human host populations of influenza viruses. The data above reveal a complex dynamic of animal to human mutation transmission patterns. g-FLUA2H provides for a detailed proteome-wide characterization of the composition and incidence of mutations present in the animal and human host populations of influenza viruses, for a better understanding of host tropism and possibly identifying human adaptation mutations that are pan-subtype or subtype-specific. This may contribute to the development of tools for surveillance of influenza viruses, and possibly to efforts for the prevention or treatment of viral infection. Given that zoonosis (human disease caused by animal pathogens) is not unique to influenza viruses, g-FLUA2H may be applicable to other zoonotic diseases, which are mostly viral origin and are emerging and re-emerging, such as rabies, Ebola, Rift valley fever, and Crimean-Congo hemorrhagic fever.

## Competing interests

We declare that we have no competing interests in this research.

## Authors' contributions

Supervised the research: JTA MAK. Conceived and designed the web application specifications: MAK MFS ST. Implemented the web application: MFS. Contributed to the content of the web application: MFS MAK ST HSAR WCL. Tested the web-application: MFS MAK ST HSAR WCL NENM. Contributed to the writing of the paper: MAK JTA MFS ST HSAR NENM WCL.

## Declaration

Publication costs for this article were funded by Perdana University.

This article has been published as part of *BMC Medical Genomics *Volume 8 Supplement 4, 2015: Joint 26th Genome Informatics Workshop and 14th International Conference on Bioinformatics: Medical genomics. The full contents of the supplement are available online at http://www.biomedcentral.com/bmcmedgenomics/supplements/8/S4.
